# Multivariate pattern classification on BOLD activation pattern induced by deep brain stimulation in motor, associative, and limbic brain networks

**DOI:** 10.1038/s41598-020-64547-7

**Published:** 2020-05-05

**Authors:** Shinho Cho, Hoon-Ki Min, Myung-Ho In, Hang Joon Jo

**Affiliations:** 1 Department of Neurosurgery, Mayo Clinic, Rochester, MN USA; 2 Center for Magnetic Resonance Research, Department of Radiology, University of Minnesota, Minnesota, MN USA; 3 Department of Physiology and Biomedical Engineering, Mayo Clinic, Rochester, MN USA; 4 Department of Radiology, Mayo Clinic, Rochester, MN USA; 5 Department of Neurology, Mayo Clinic, Rochester, MN USA; 6 Department of Physiology, College of Medicine, Hanyang University, Seoul, South Korea

**Keywords:** Network models, Neural circuits

## Abstract

Deep brain stimulation (DBS) has been shown to be an effective treatment for movement disorders and it is now being extended to the treatment of psychiatric disorders. Functional magnetic resonance imaging (fMRI) studies indicate that DBS stimulation targets dependent brain network effects, in networks that respond to stimulation. Characterizing these patterns is crucial for linking DBS-induced therapeutic and adverse effects. Conventional DBS-fMRI, however, lacks the sensitivity needed for decoding multidimensional information such as spatially diffuse patterns. We report here on the use of a multivariate pattern analysis (MVPA) to demonstrate that stimulation of three DBS targets (STN, subthalamic nucleus; GPi, globus pallidus internus; NAc, nucleus accumbens) evoked a sufficiently distinctive blood-oxygen-level-dependent (BOLD) activation in swine brain. The findings indicate that STN and GPi evoke a similar motor network pattern, while NAc shows a districted associative and limbic pattern. The findings show that MVPA could be effectively applied to overlapping or sparse BOLD patterns which are often found in DBS. Future applications are expected employ MVPA fMRI to identify the proper stimulation target dependent brain circuitry for a DBS outcome.

## Introduction

While deep brain stimulation (DBS) has been an effective treatment for alleviating motor-related symptoms, i.e., Parkinson’s disease^1,2^, applications have been further extended to neuropsychological disorders, i.e., obsessive-compulsive disorder (OCD)^3^. The pathological transmission in local neuronal populations can be inhibited by DBS (‘jamming effect’)^4^. It is also evident that the DBS effect would induce broadly diffused activation across brain networks (‘network effect’)^5–7^. The mechanism of DBS action has been extensively investigated at the cellular and local circuitry levels, but it remains unclear to what extent distinctive effects in a whole brain network might occur when different regions of the brain are electrically stimulated.

Due to its non-invasive and large coverage of imaging, functional magnetic resonance imaging (fMRI) is suitable for mapping characteristic DBS effects^8,9^. However, DBS-induced fMRI BOLD (blood-oxygen-level dependent) responses in general would be expected to appear across brain regions in the form of spatially overlapping and complicated patterns, making it difficult to characterize the unique modulatory pattern for a given DBS. Notably, cumulative DBS studies have reported that distinctive targets could induce similar therapeutic effects; in contrast, the same targets could result in different types of side effects^2,10,11^. Therefore, disentangling whole brain BOLD patterns and correlating their relationships with clinical outcomes in DBS studies is a crucial issue.

Conventional “univariate” analyses for fMRI are insensitive for differentiating BOLD spread patterns^12^, particularly for patterns that are sparse and overlapping across wide networks. In this case, the similarity and difference in collective responses in multiple ROIs could be overlooked^13^. In contrast, recent multivariate pattern analyses (MVPA)^14–16^ have demonstrated the presence of discrete BOLD patterns, that reflect differing visual tasks^17^, and emotional states^18^, and also in clinical studies, i.e., the diagnosis of depression^19^, heroin dependency^20^, and symptom severity predictions in cases of autism^21^. Thus, MVPA has now become more popular and shows superior performance for differentiating overlapping or sparse BOLD patterns^22^. Unlike the conventional analysis of variance (e.g., t-test) using the average for a large ROI, MVPA utilizes the feature vector (collective representation of multiple ROI or voxel response), in which individual responses are merged into a multidimensional vector.

**Figure 1:**
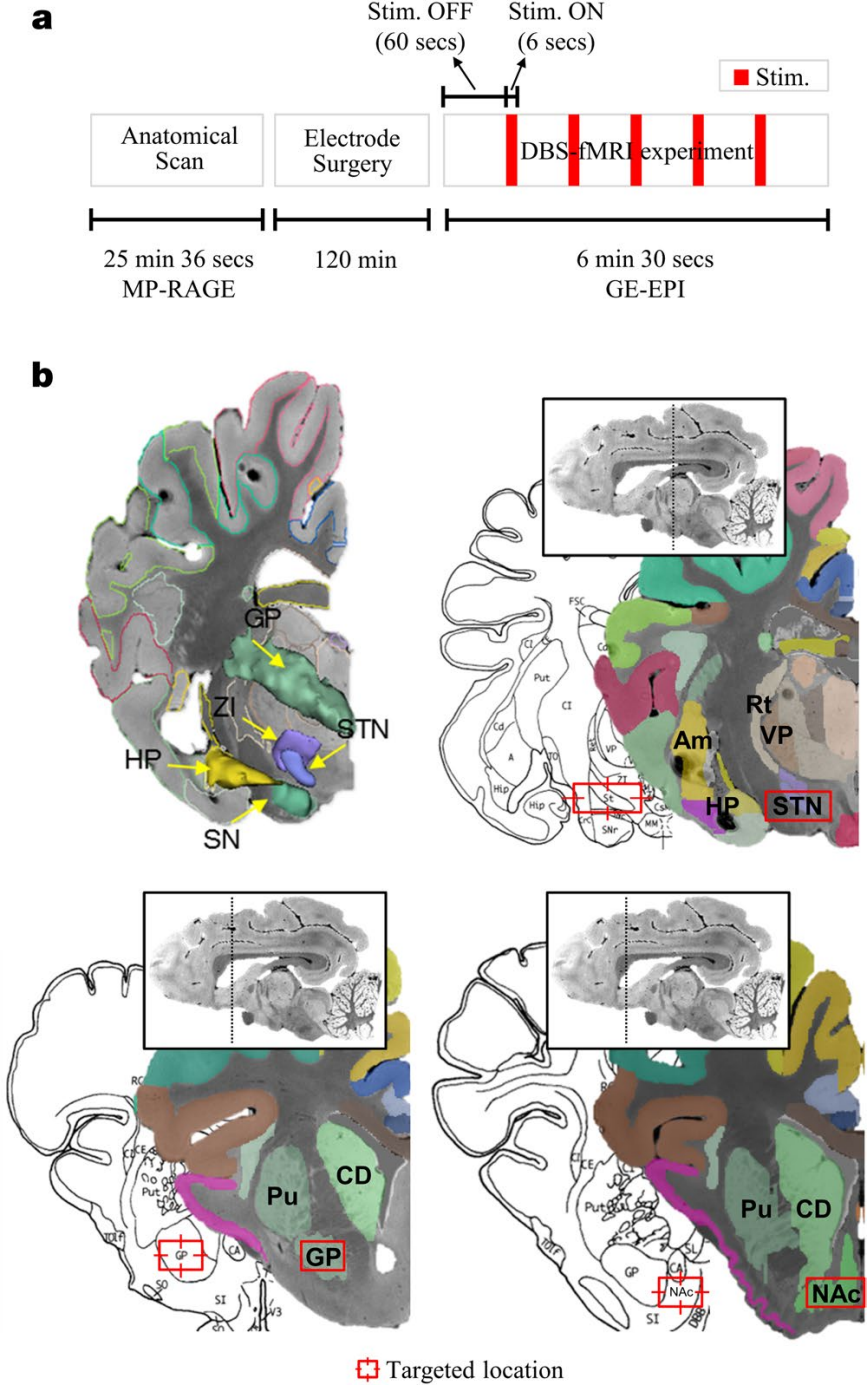
(**a**) Schematic diagram depicting the experimental procedure and (**b**) the anatomical location of target in DBS groups (STN, GPi, and NAc) overlaid on the pig brain atlas^57,58^. Abbreviations: GE-EPI, gradient-echo echo planar imaging; GPi, globus pallidus interna; STN, subthalamic nucleus; NAc, nucleus accumbens; MP-RAGE, magnetization prepared rapid gradient echo.

**Figure 2:**
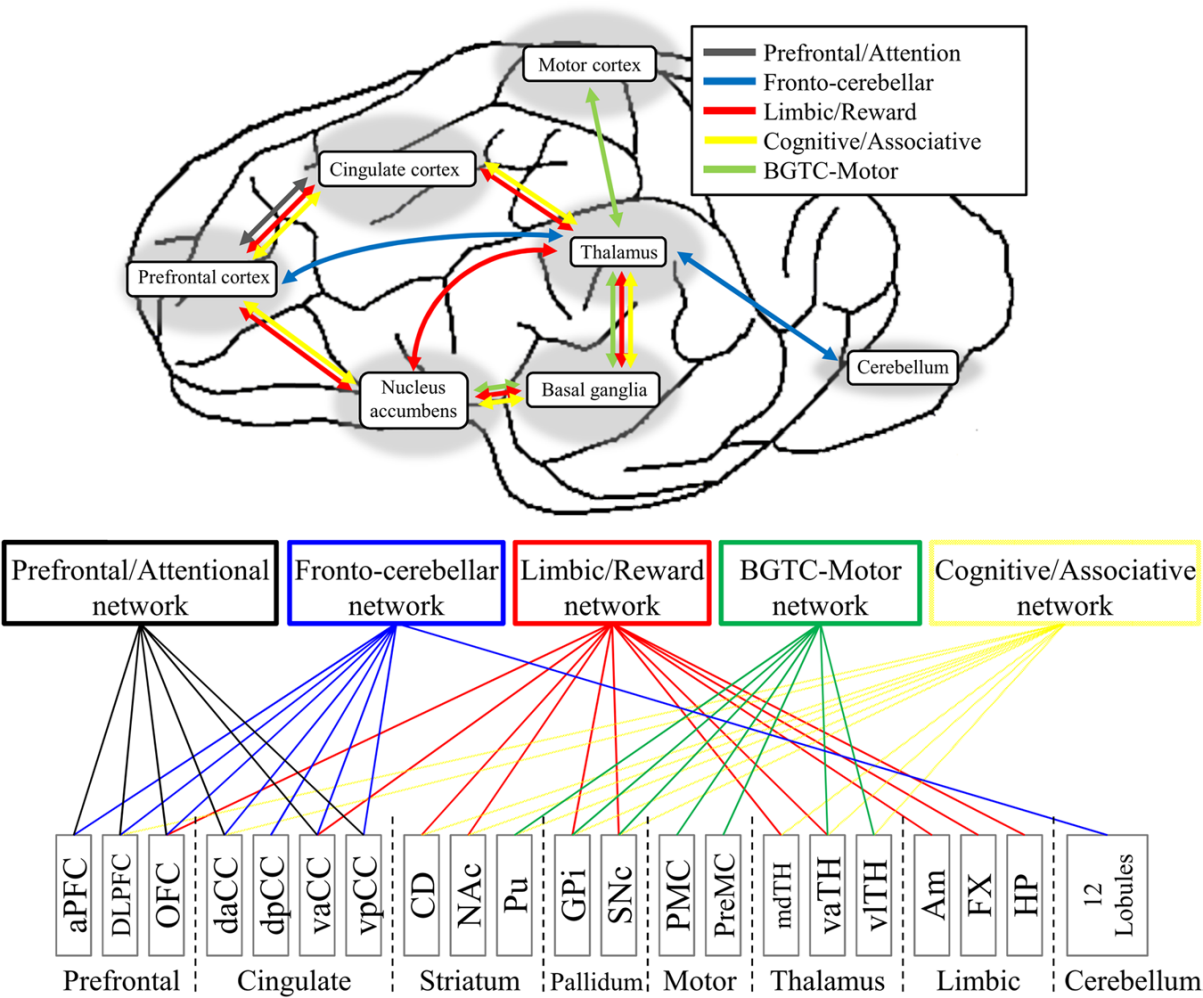
Schematic diagram for functional networks and categorization of the individual region-of-interests (ROIs) to network-of-interests (NOIs). Five networks are represented in distinctive colors; the Prefrontal/Attention (gray) network^61,65^, the Fronto-cerebellar network (blue)^63,74^, the Limbic/Reward circuit (red)^65^, the basal-ganglia-thalamocortical (BGTC) motor network (green)^59,60^, and the Cognitive/Associative network (yellow)^61,62^. The figures were created by See for abbreviations in Appendix A.

Here we examined the distinctive network-wide, BOLD modulation patterns that were induced by three DBS targets (STN, GPi, and NAc; for abbreviations, see Appendix A) in swine (total 21 subjects, seven in each group) using the multivariate pattern classification analysis (MVPA). Figure 1 depicts the locations of each DBS target. We systematically expanded the region-of-interests (ROIs) to network-of-interests (NOIs) that represents whole-brain functional networks (Fig. 2). An inter-group pattern classification was then conducted to assess the similarity and difference in BOLD activation across differing DBS targets; with particular emphasis on five, disease-related functional networks with clinical implications for DBS.

## Results

### Group-level BOLD activation of STN, GPi, and NAc stimulation

**Figure 3:**
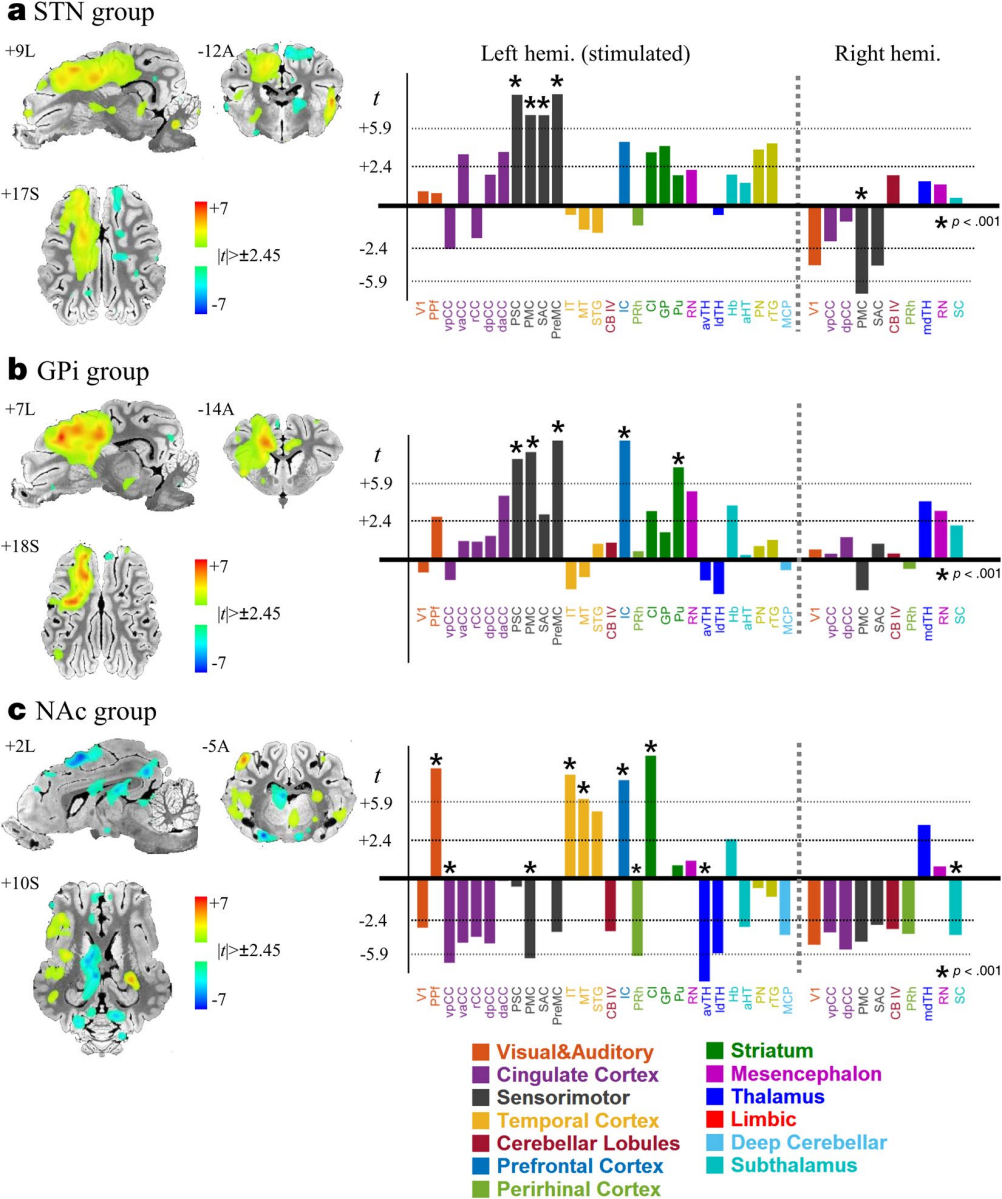
Group-level BOLD activation map and *t*-statistics (*n* = 7 per group); (**a**) STN DBS, (**b**) GPi DBS, and (**c**) NAc DBS group. The activation map shows the significant BOLD activation (one-sample two-tailed *t*-test, *t*[6]> 2.45, *p* < 0.05, False discovery rate [FDR] corrected). Multiple cortical and subcortical brain regions on the ipsilateral (left) hemisphere evoked significant responses. The bar graphs show the *t*-statistics for the regions with asterisks indicating *p* < 0.001 (*t*[6]> 5.9, FDR corrected). See for abbreviations in Appendix A. Data of all brain regions are shown in Fig. S3.

Figure 3 shows the significant BOLD activations that were identified in multiple cortical and subcortical areas in each stimulation group (*p* < 0.05; *t* > 2.45, FDR corrected). STN stimulation evoked activations in the ipsilateral (left) PMC, PSC, Pu, and in the contralateral (right) aPFC, IC, and PPf (Fig. 3a). GPi DBS, similar to the STN DBS, evoked significant activations in ipsilateral PMC, PSC and PreMC (Fig. 3b). While both STN and GPi DBS commonly activated sensorimotor regions, it should be noted that additional activations were observed only in the GPi group, i.e., dpCC, daCC, DLPFC, and Pu. Interestingly, the STN DBS group induced significantly decreased activations in the contralateral PMC, while GPi DBS did not, indicating that the DBS effect may be lateralized across hemispheres.

**Figure 4:**
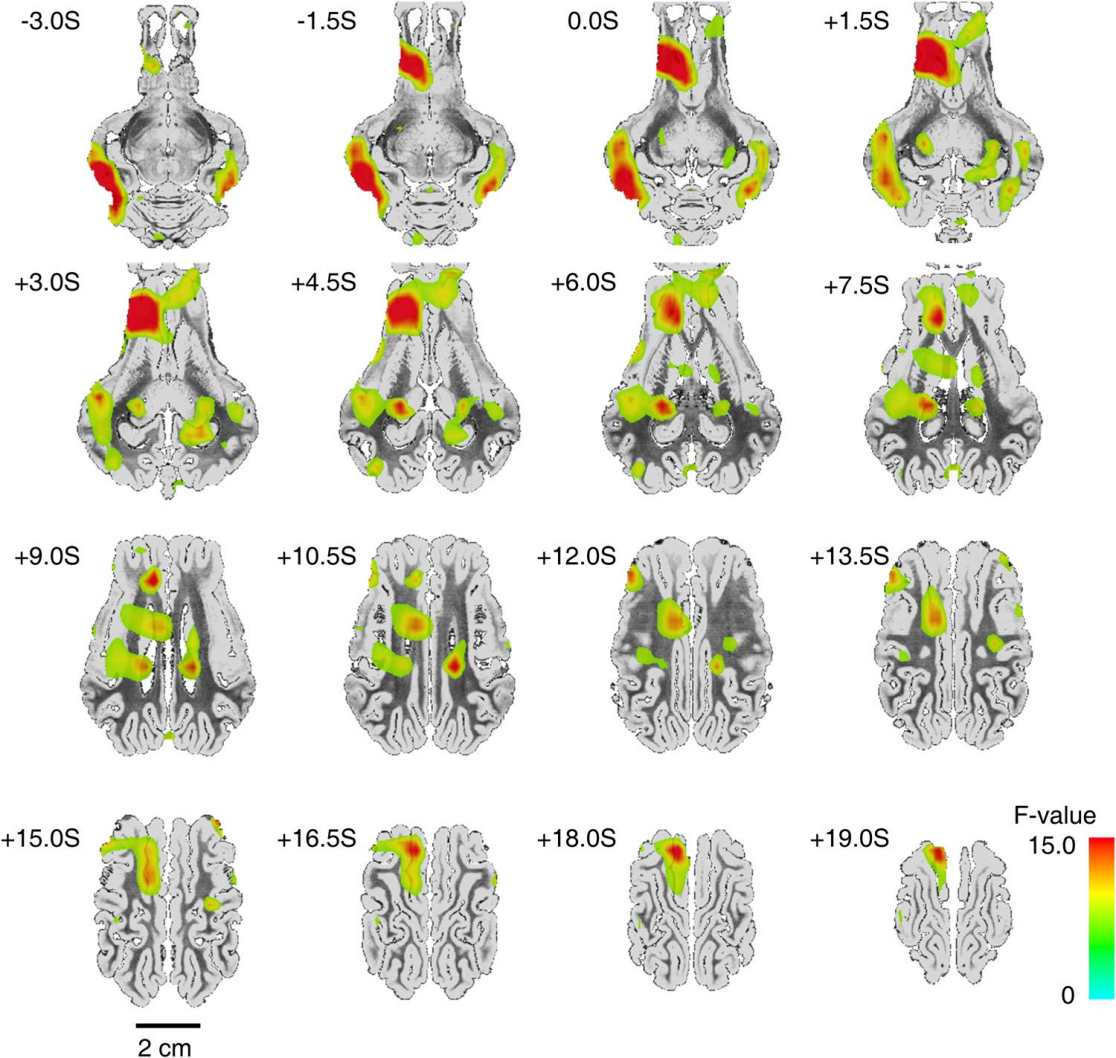
Group-level BOLD activation map of the analysis of variance (ANOVA) (3 groups × 7 subjects). Significant group differences in activation were detected in several cortical (sensorimotor, prefrontal cortex, and cingulate cortex), subcortical (thalamic subregions, basal ganglia complex) areas, and the cerebellum (F[2,6]> 5.14, *p* < 0.05). For the detailed group-wise comparison, see Fig. S4. See for abbreviations in Appendix A.

Unlike the two groups, NAc DBS showed a mixed effect; some areas showed significantly increased BOLD responses (IC, Cl and PPf), but also decreased activity (ipsilateral PMC and avTh) (Fig. 3c). Overall STN and GPi stimulation appears to have a similar modulatory effect on motor and motor-related systems, whereas NAc stimulation has a greater impact on non-motor systems, primarily concentrating on the reward-related subcortical areas. For further detailed comparisons, see Fig. S4. Additionally, two-way ANOVA results (F[2,6]> 5.14, p < 0.05) indicated that three DBS targets evoked distinctive patterns in the ipsilateral cortical (prefrontal, sensorimotor, and cingulate cortex) and subcortical brain areas (thalamus, Cd, GPi, Pu, Hp, RN) (Fig. 4). For abbreviations, see Appendix A.

### Inter-group comparison of cortex-level BOLD activation pattern

**Figure 5:**
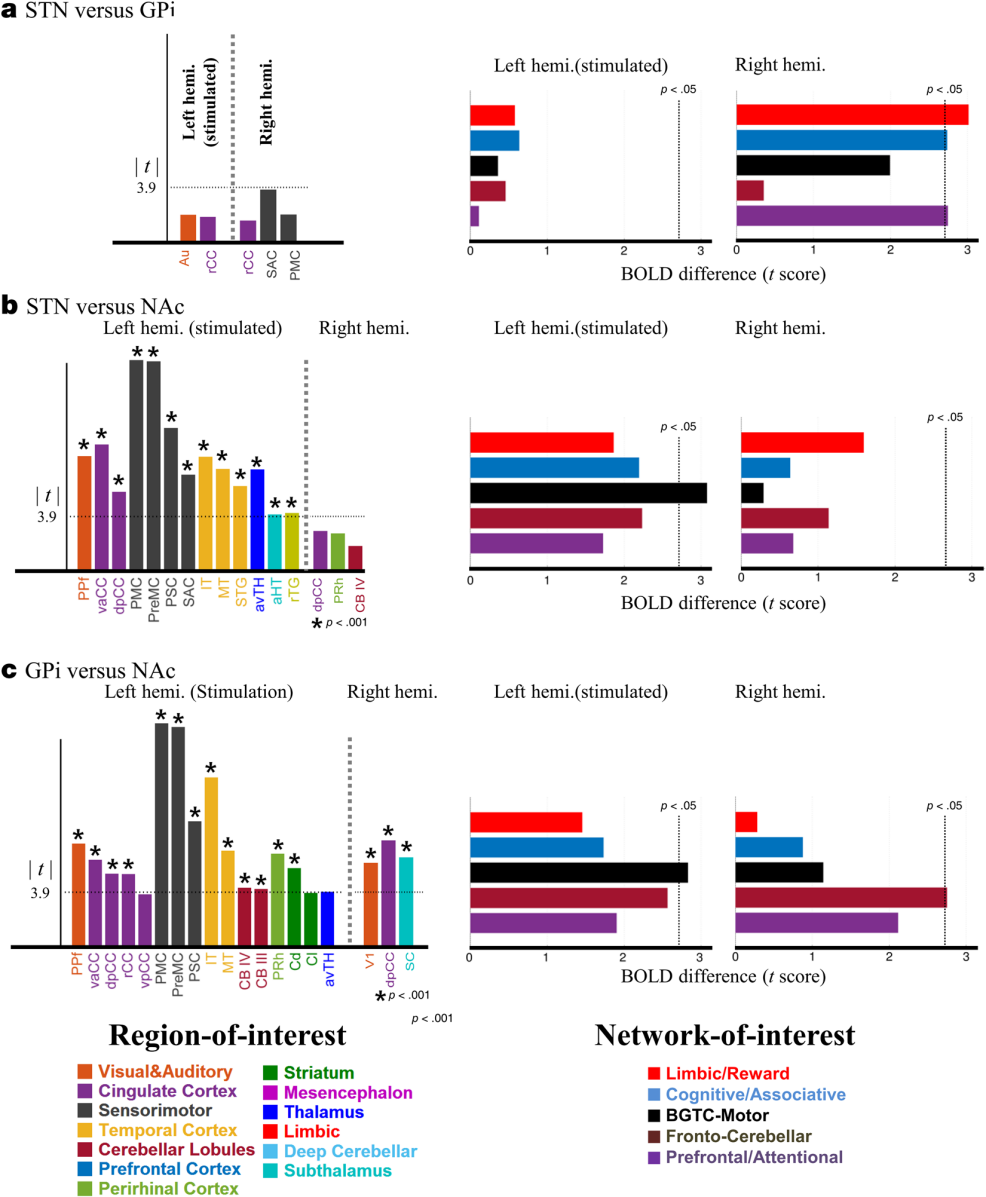
Inter-group comparison of BOLD response at the cortex-level of regions of interests (ROIs) (left) and network-of-interests (NOIs) (right). T-statistics of mean difference in averaged ROI and NOI response were shown by different color bars; (a) STN versus GPi, (**b**) STN versus NAc, and (**c**) GPi versus NAc DBS group **(**two-sample, two tailed *t-*test**)**. Few regions evoked significant differences between the STN and GPi groups, while the overall activation was sufficiently different in the NAc group from that of the STN and the GPi DBS group (*p* < 0.001 (*t*[12]> 3.9, corrected). Further network-level analysis (right) shows that significant differences presented in the contralateral networks (limbic, associative, and prefrontal networks) in STN vs. GPi group. Three ipsilateral networks (Cognitive/associative, BGTC-motor, and Fronto-cerebellar) and two networks (BGTC-motor and Fronto-cerebellar) evoked sufficiently different activations (*p* < 0.05) between STN and NAc and between GPi and NAc DBS group respectively. See for abbreviations in Appendix A.

A cortical level inter-group comparison of BOLD response patterns (Fig. 5, left) indicated that there was no significant difference between the STN- and GPi-DBS groups (Fig. 5a). In contrast, in many regions, the BOLD modulatory patterns were different between the STN and NAc groups (Fig. 5b), and between the GPi and NAc groups (Fig. 5c); clear differences in sensorimotor and reward-related areas were observed (*t*[12]> 3.9, *p* < 0.001, corrected). The overall results indicate that STN and GPi DBS could evoke less distinguishable effects based on the statistics for BOLD activation, whereas the effects observed in STN and GPi DBS appear to be sufficiently different from that of NAc DBS. It should be noted that voxel-wise activation mapping indicated that each STN and GPi group has its own characteristic pattern, i.e., SAC. However, a group comparison based on a two-sample variance test (*t*-test) failed to detect significant differences, indicating that the sensitivity of discrimination would be decreased due to the broad extent of a ROI.

### Inter-group comparison of network-level BOLD activation pattern

A further network-level inter-group comparison delineated the BOLD activation differences among the three DBS groups (Fig. 5). While an analysis of variance test (*t*-test) in the cortex-level failed to detect any significant differences between the STN and GPi groups, the network-level comparison showed that the contralateral hemisphere evoked a distinctive activation pattern (*t*[12]> 2.17; *p* < 0.05) (Fig. 5a), which was particularly pronounced in the reward-related and cognitive networks. This suggests that the characteristic effect STN DBS modulates bilaterally, whereas GPi DBS only impacts the ipsilateral hemisphere. For the NAc group, in general agreement with the cortical level comparisons, the ipsilateral BGTC-Motor (basal ganglia-thalamocortical-motor) and Fronto-Cerebellar networks showed significantly different activations from those in the STN and GPi group (Fig. 5b,c).

### Inter-group multivariate pattern classification of network-level BOLD activation pattern

**Figure 6:**
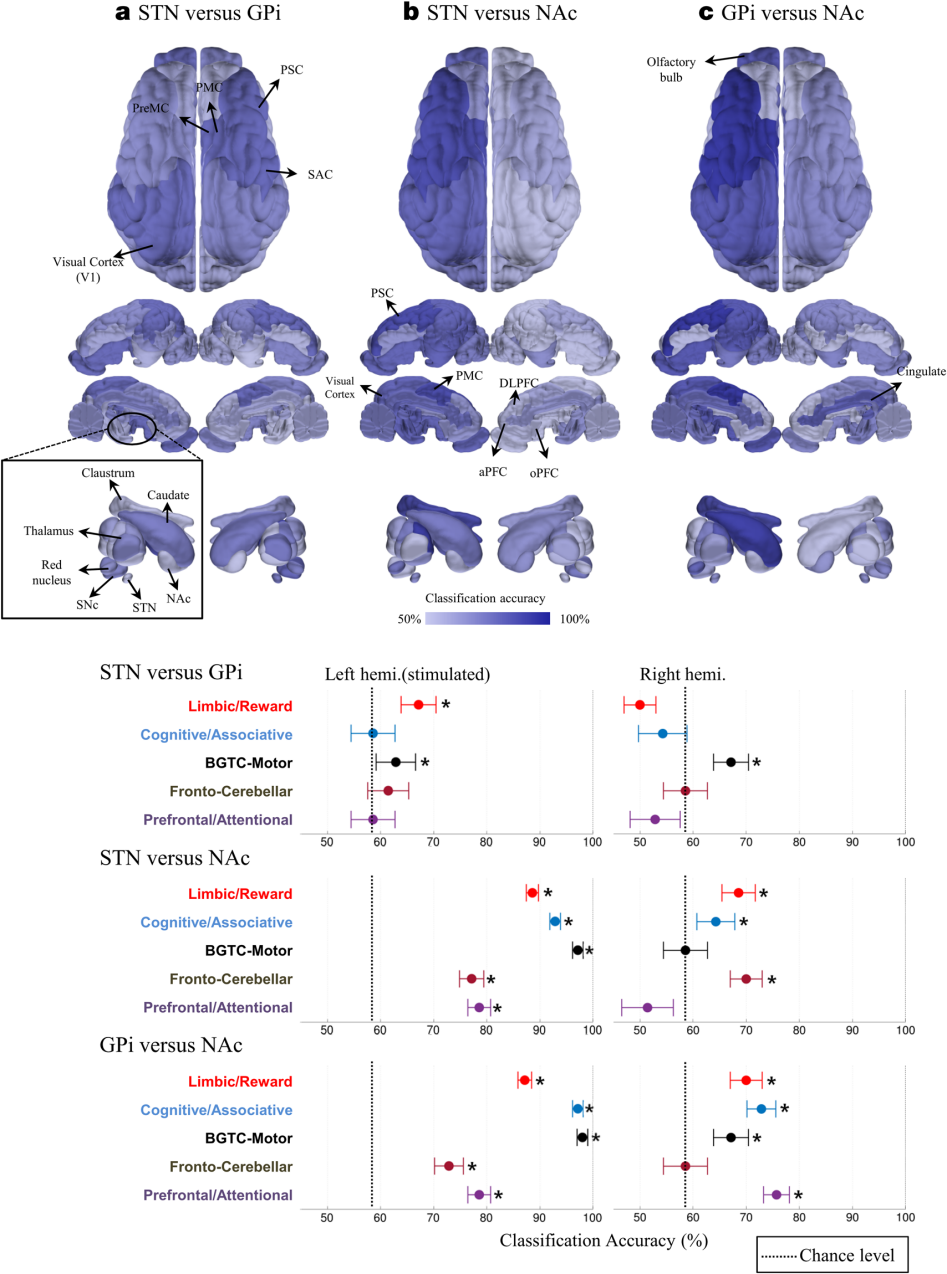
Inter-group multivariate pattern classification results at five functional networks. The accuracy of pair-wise classification between groups (true-positive rate) was represented by differing color intensities on a 3D pig brain model (top) and graphs (bottom); (**a**) STN DBS versus GPi DBS, (**b**) STN versus NAc group, and **(c**) GPi versus NAc group. Filled circles indicate the accuracy rate (error bars denotes ±1 standard error of the mean). The vertical dashed line indicates the bias-corrected chance level (59.3%). Asterisks indicate classification accuracy that exceeds the chance level. Distinctive network patterns were detected in the ipsilateral limbic and both ipsilateral and contralateral motor networks between STN and GPi DBS group. NAc DBS evoked substantially distinctive BOLD response pattern in many networks; highly distinctive network-wide pattern presents in the ipsilateral sensorimotor system and cognitive/associative networks including multiple subcortical areas. 3D brain images were rendered by Matlab R2015a software (The MathWorks, Inc., Natick, MI, USA) based on the pig brain atlas^57,58^. Abbreviation: BGTC-Motor, basal ganglia-thalamocortical-motor; see Appendix A for the abbreviations.

Lastly, we expanded our scope to a comparison of large-scale whole brain networks and applied the multivariate pattern classification. The classification results revealed the presence of clearly and significantly distinguishable patterns in five networks among the groups (Fig. 6). STN and GPi DBS induced a significant, distinct pattern in ipsilateral Limbic/Reward (classification accuracy, true positive: 67.14 ± 3.29%, ±1 standard error of the mean), and bilateral BGTC-Motor (basal ganglia-thalamocortical-motor) networks (62.86 ± 3.71% for the left hemisphere and 67.14 ± 3.20% for the right hemisphere) (Fig. 6a), in which all of the accuracy was significantly higher than the chance-level rate (59.8%, *p* < 0.05, bias-corrected). Interestingly, the pattern in the contralateral hemisphere was distinctive for three networks, in which variance tests found no differences; Limbic/Reward (68.57 ± 3.14%), Cognitive/Associative (64.29 ± 3.57%), and Fronto-Cerebellar network (70.07 ± 3.02%).The STN and NAc group were distinctive in all five ipsilateral networks (Fig. 6b); Limbic/Reward (88.57 ± 1.14%), Cognitive/Associative (92.86 ± 0.71%), BGTC-Motor (97.14 ± 0.29%), Fronto-Cerebellar (77.14 ± 2.29), and Prefrontal/Attentional network (78.57 ± 2.14%). A similar classification performance was achieved between the GPi and NAc groups (Fig. 6c); Limbic/Reward (87.14 ± 1.29%), Cognitive/Associative (87.14 ± 1.29%), BGTC-Motor (98.14 ± 1.00%), and Cognitive/Associate network (97.0 ± 0.29%) including Fronto-Cerebellar (72.86 ± 2.71%) and Prefrontal/Attentional network (78.57 ± 2.14%).

## Discussion

The characteristic network-wide activation pattern induced by DBS reflects its clinical effectiveness^6^. STN and GPi DBS are generally thought to inhibit the pathological oscillations within the BGTC (basal ganglia-thalamocortical) motor network, thus alleviating motor-related symptoms. In our results, STN and GPi DBS commonly evoked network-wide activations across sensorimotor regions including stimulation sites. These results are consistent with previous DBS-fMRI reports, in which those corresponding areas evoked BOLD responses by STN/GPi DBS^23^ and STN DBS^24^, but further activations were also detected in the thalamic subregions, SNc, SC in the STN DBS^25^. Positron emission tomography (PET) results were overall similar to the fMRI results; i.e., STN DBS induced regional CBF (cerebral blood flow) and an increase in metabolic rate at the stimulation site (STN) and thalamic subregions. However, contrasting results were also found, in that the metabolic rate was actually decreased in some areas, i.e., GPi, Pu, and the sensorimotor cortex^26,27^. One possibility for this would be that differing stimulation parameters (voltage and frequency) may have caused the decreases^27^. Alternatively, the location of the variances of stimulations could have influenced the consistency of the results^28^.

While few functional imaging studies examined the similarity and difference in functional BOLD activation, i.e., STN and GPi DBS comparison^23^, cumulative clinical reports have pointed to distinctive behavioral effects being associated with those targets. It is generally accepted that both STN and GPI DBS are effective for alleviating motor function-related symptoms, but STN DBS has the potential for also being helpful in reducing psychiatric symptoms, i.e., the severity of OCD^3,29^ and the dose of levodopa medication^30^ as shown by levodopa-PET results^26^. Moreover, some studies showed that STN DBS could be associated with undesirable cognitive effects, i.e., mania or depression^31–33^, while cognitive effects have not been reported from GPi DBS group. Our MVPA classification showed that STN and GPi DBS has a discintive network-wide BOLD pattern in the limbic system, providing an insight for understanding the psychiatric effect of STN DBS.

The modulating effect of STN and NAc DBS were sufficiently distinctive. While the strongest modulation of NAc DBS appeared in the prefrontal cortex, previous behavioral studies suggested the possibility that NAc stimulation could partially modulate motor functions shown from animal studies, i.e., suppressed leg flexion^34^ and vocalization^35^. However, our data did not directly confirm this possibility, since STN and NAc still evoked distinctive network patterns in the motor cortex. Some reports, however, have indicated that NAc DBS can be effective for treating the motor tick of Tourette syndrome^36^, albeit these findings are still experimental. Rather, our results show that NAc DBS has a mixed effect; BOLD responses could be either facilitated or suppressed depending on the brain region, as has been previously reported in applications of NAc-DBS for patients^28^. This is likely due to complex afferent and efferent projections of NAc to many striatal substructures^37^, suggesting subtle difference in stimulation foci may be influential for network modulatory pattern.

Using swine would be beneficial for translational research in DBS-fMRI. The brain volume of the swine (~160 g) is larger than that of rodents, comparable to the non-human primate brain (e.g., the rhesus monkey)^38^, because a gyrencephalic neocortex and subcortical structures more closely represents the human cerebral cortex^39^. However, because functional structures related to the higher cognitive function would be expected to be different between the swine and the human brain, our results should be carefully interpreted in terms of their translatability to humans.

Several limitations should be also considered. First, the present study does not provide behavioral correlations with DBS-induced activation patterns. Thus the validity of MVPA for differentiating activation patterns needs to be justified by behavioral changes associated with each DBS target. Moreover, DBS-evoked brain responses may not fully represent the response of a disease-state brain, since healthy animals were used in the present study. Alternatively, recent animal disease-model studies have been progressing, swine PD^40^ and MPTP PD model (1-methyl-4-phenyl-1,2,3,6-tetrahydropyridineis) in squirrel monkey^41^. Although some factors would make it difficult to generalize the present results for a therapeutic strategy, future studies should be able to validate MVPA approach for the eventual translation to human DBS. For example, the brain patterns of therapeutic stimulation can be differentiated from those in ineffective stimulations. This could eventually enable us to characterize the brain pattern related to optimal DBS outcomes.

Second, anesthesia could bias the interpretation of BOLD activation. It has been reported that the depth of anesthesia^42^ influences the amplitude of the BOLD signal change, and that a substantial reduction in BOLD responses is observed in anesthetized patients^43^. Deep anesthesia can induce burst suppression^44^, which can influence the dynamics of cerebral blood flow (CBF)^42^ that can also cause changes in BOLD signals. While a higher concentration (>1.8%) would more likely evoke burst suppression, the issue of the specific dose needed to induce burst suppression in differing species in not clear^45^. In general, isoflurane (<1.5%) tends to reduce the BOLD response; therefore, the degree of activation in the present study might be underestimates rather than overestimates. Additionally, it also should be considered that different brain areas would be affected differently by isoflurane, i.e., a certain region might be more susceptible than others^46^. All those possibilities suggest that the results would be less generalizable for the awaked state. However, we confirmed that the present DBS-fMRI paradigm including stimulation parameters were quite robust and evoked detectable BOLD responses across different trials and subjects^23,47–49^. Yet the impact of anesthesia on BOLD responses should not be overlooked.

Third, the use of different stimulation parameters could alter the extent of diffused BOLD activation. Unlike chronic DBS, an acute 6-sec stimulation paradigm was adopted in the present study. Constant stimulation for a prolonged amount of time could alter the BOLD activation pattern, because it cannot be ruled out that stimulation could alter network connectivity in a brain. In addition to the duration of stimulation, a higher voltage tends to evoke a more widely spread BOLD activation^48,49^. Thus the reproducibility of MVPA classification could be influenced by the experimental setting. However, the *relative* activation differences between ROIs within a single network may be preserved, even if the spatial extent and degree of activation could vary. Because individual ROIs’ responses were normalized first and then fed into MVPA as a feature vector, we conclude that MVPA performance would remain robust. Additionally, we did not perform a pre-surgical fMRI; therefore, the issue of whether or not the intrinsic functional connectivity influences the BOLD pattern remains inconclusive. A previous study showed that the stronger is the functional connectivity between ROIs, the more likely are the regions to co-activate^50^, suggesting that the latent connectivity could systematically skew MVPA classification accuracy.

Lastly, metal components of a DBS electrode can result in the production of magnetic susceptibility artifacts (Fig. S5), in which the MR signal is significantly reduced near the implantation site, resulted in the BOLD activation being less detectable. While spin-echo EPI is less vulnerable for susceptibility artefacts, the application can be limited due to the slow acquisition time and heating^51^. Alternatively, the recent development of zero echo time fMRI techniques (e.g., Multi-band SWIFT)^52,53^ or Point-spread-function-based phase distortion correction^54^ would be promising in terms of reducing susceptibility-induced artifacts for a metallic electrode.

## Materials and Methods

### Animal preparation

DBS electrodes were implanted in the left hemisphere of 21 healthy swine (three groups; the subthalamic nucleus (STN), globus pallidus interna (GPi), and nucleus accumbens (NAc); *n* = 7 per each target group). Each animal (8–12 months old, 25–30 kg body weight) was initially sedated by an intramuscular injection of a Ketamine (15 mg/kg), xylazine (2.5 mg/kg), and Telazol (5 mg/kg) cocktail and then mechanically ventilated through an orally intubated tube (respiration cycle: 12 per minute). Anesthesia was maintained by a 7:3 of N_2_O:O_2_ medical gas mixture with isoflurane (1.5–2.0% during DBS surgery and 1.3–1.5% during anatomical/functional image acquisition). Animal physiology was monitored by pulse oximetry, rectal thermometry, and capnography sensor (Nonin Medical Inc, MN) and maintained at normal conditions (heart rate: ~120 bpm, rectal temperature: 37 ± 1 °C, spO_2_: 98~100%, and end-tidal CO_2_: 3.5~4%). The study procedures were performed in accordance with the National Institutes of Health Guidelines for Animal Research. The Mayo Clinic Institutional Animal Care and Use Committee (IACUC) approved the present experimental protocol.

### DBS implantation

The DBS target is depicted in Fig. 1b and locations of electrode tip are shown in Fig. S1. For DBS implantation, anatomical 3D MP-RAGE imaging was conducted on a General Electric Signa HDx 3.0 Tesla scanner with following parameters: repetition time (TR) = 11 ms, echo time (TE) = 5.16 ms, field of view (FOV) = 24 × 24 (mm), matrix size = 256 × 256, slice number = 128, 4-channel custom-built radio frequency (RF) coil. By using the COMPASS stereotactic planning software (COMPASS International Innovations, Rochester, MN), three coordinates (arc, collar, and depth) were identified for each anatomical target (Fig. 1b), and imported into the Leksell stereotaxic system (Elekta, Stockholm, Sweden) based on the individual subject’s anatomical image. A micro drive system (Alpha-Omega system, Co. LTD., MN) guided the Model 3389 DBS electrode (Medtronic Inc.) to the prepared coordinate. The location of the DBS electrode was visually confirmed through MR-CT fusion images after each surgery^23^. For visualization of DBS targets, subcortical structures (STN and GPi) were rendered in three dimensional shape by 3D Slicer^55,56^ and overlaid on the pig brain atlas^57,58^.

### fMRI data acquisition

We used block design of DBS-fMRI (five DBS stimulation and following rest periods) (Fig. 1a). Stimulation was conducted with following parameters; a gradient-echo echo-planar imaging (GE-EPI); TR = 3000 ms, TE = 34.1, flip angle (FA) = 90°, slice thickness = 2.4 mm, FOV = 150 ×150 mm, matrix size = 64 ×64, total acquisition time was 6 minutes 30 seconds.

### Stimulation parameters

The stimulation parameters were as follows: bi-phasic mono-polar pulse train; voltage, 5 v; pulse frequency, 130 Hz; pulse width, 90 μs with the two of the four contacts on the lead, 0 (anode) and 1 (cathode). The voltage and frequency setting were determined based on our previous results that the parameters induced the robust and reproducible BOLD activation across trials^23,47–49^.

### Region-of-interests (ROIs) generation

We first divided the brain area into 73 regions (for each hemisphere) according to the anatomically labeled pig atlas^58^. Figure 2 shows the affiliation of an individual brain region to cortices/lobules and networks. Functional networks definition in the present study were based on the previous literatures: the limbic/reward network;^59,60^ the cognitive/associative loop;^61,62^ the basal-ganglia-thalamocortical (BGTC) motor circuit^59^; the fronto-cerebellar network^63^, and the prefrontal/attentional network^64,65^. Due to a metallic DBS lead caused susceptibility artifact, GE-EPI BOLD signals substantially decreased near an electrode lead. The time series of those voxels were excluded in our analysis.

### Functional imaging data preprocessing

All analyses were performed using the AFNI software (Analysis of Functional NeuroImages)^66^. Individual EPI volumes were preprocessed as following orders: spike removal, slice-timing correction, and motion correction (X, Y, Z, yaw, pitch, and roll). Then the individual image volumes were co-registered to the pig brain atlas^58^ by the local Pearson’s correlation^67^. Spatial smoothing (3 mm Gaussian Kernel Full-width-half-maximum) and temporal filtering (band-pass filtering between 0.01 and 0.15 Hz) were applied.

### Univariate statistical analysis

By applying General linear modeling (GLM), statistical mapping of BOLD activation was conducted and beta coefficients and their *t* statistics were estimated. The baseline drift (six orders) and motion-induced artefacts were removed prior to GLM analysis. To obtain a group-level activation map, the beta coefficients were averaged per group. A statistical threshold for beta coefficient and the Gaussian-based cluster correction (3dClustsim in AFNI software) were used to remove false positive activation and identify significant activation clusters (*p* < 0.05, false discovery rate [FDR] corrected). In cortical and network-level analysis, we applied the singular vector decomposition (SVD) for a group of voxels in ROIs and NOIs^68,69^, in which time series were effectively averaged with the reduced bias in time series. Then the analysis of variance (*t*-test and two-way ANOVA) was carried out in cortex-level ROIs (regions-of-interests) and network-level NOIs (network-of-interests) between groups; STN versus GPi, STN versus NAc, and GPi versus NAc DBS group in three levels of ROI (NOI); regional, cortical, and network level. In a two-way ANOVA analysis, the group effect was tested (3 groups × 7 subjects), in which the individual effect set to the random variable.

### Multivariate Pattern Classification for network-level analysis

To differentiate network level BOLD patterns between groups, multivariate pattern analysis (MVPA) was used with the Fisher’s LDA (linear discriminant model)^70^. A custom-built Matlab code was made by combining multiple components from built-in functions and the statistical toolbox in Matlab (Matlab R2015a, The MathWorks, Inc., Natick, MI, USA), and functions from the Princeton Multi-Voxel Pattern Analysis toolbox^71^. First, we assigned the beta coefficients estimated from regional BOLD activation into a multi-dimensional feature vector, wherein a single feature vector represents the DBS response of a network-of-interest (NOI) (Fig. 2). The feature vectors (7 subjects × 5 stimulation blocks × 2 comparing groups) were then randomly partitioned to the equally sized bins (*k* = 10) with a group label (STN, GPi, or NAc). Then training data sets (*k*-1) were fed into the LDA (Linear Discriminant analysis) for training and remaining one block was used for testing the trained model. To validate the classification results, a k-fold repeated cross-validation procedure with a random resampling^72^ was adopted; the classification was repeated over 1000 times in conjunction with a random resampling method in each comparison (STN vs. GPi, STN vs. NAc, and GPi vs. NAc). The true-positive classification rate was then calculated and a one-sample *t*-test was applied on the mean and standard deviation of correct classification to obtain the statistical significance. The procedure was repeated for three pair-wise groups in each of five functional networks (network-of-interests). We used the sample size-bias corrected chance level, which is 59.8% at *p* < 0.05, because the empirical chance level varies depending on the sample size^73^.

## Supplementary information

Supplementary information.

## APPENDIX A: Abbreviations for anatomical labels: Abbreviations for anatomical labels

APPENDIX A:

aPFC, anterior prefrontal cortex

Am, amygdala

Au, auditory cortex

CB III, cerebellar lobule III

CB IV, cerebellar lobule IV

daCC, dorsal anterior cingulate cortex

dpCC, dorsal posterior cingulate cortex

rCC, retrosplenial cingulate cortex

vaCC, ventral anterior cingulate cortex

Cd, caudate nucleus

Cl, claustrum

DLPFC, dorsal lateral prefrontal cortex

FX, fornix

GP, globus pallidus

GPi, globus pallidus interna

Hb, habenular nuclei

HP, hippocampus

aHT, anterior hypothalamic area

IT, interior temporal gyrus

IC, insular cortex

MCP, medial cerebellar peduncle

MT, middle temporal gyrus

NAc, accumbens nucleus

OFC, orbitofrontal cortex

PreMC, premotor cortex

PMC, primary motor cortex

PN, pontine nucleus

PPf, prepiriform area

PRh, perirhinal cortex

PSC, primary somatosensory cortex

Pu, putamen

RN, red nucleus

Rt, reticular thalamic nucleus

SAC, sensory association cortex

SC, superior colliculus

SNc, substantia nigra pars compacta

STG, superior temporal gyrus

rTG, renticulotegmental nucleus

avTh, anterior ventral thalamic nucleus

ldTh, lateral dorsal thalamic nucleus

mdTh, mediodorsal thalamic nucleus

rTh, reticular thalamic nucleus

vaTh, ventral anterior thalamic nucleus

vpCC, ventral posterior cingulate cortex

vlTh, ventral lateral thalamic nucleus

vmTh, ventral intermediate thalamic nucleus

V1, primary visual cortex
