# Supplementary material for: Multivariate pattern classification on BOLD activation pattern induced by deep brain stimulation in motor, associative, and limbic brain networks
Source: Sci Rep. 2020 May 5;10:7528. doi: 10.1038/s41598-020-64547-7 (PMC7200672; doi:10.1038/s41598-020-64547-7)
Supplement: Supplementary file 1 — Supplementary information. [file 41598_2020_64547_MOESM1_ESM.pdf]

Multivariate pattern classification on BOLD activation pattern induced by deep brain stimulation in motor, associative, and limbic brain networks

Shinho Cho[1,2], Hoon-Ki Min[1,3,4], Myung-Ho In[1,4], and Hang Joon Jo[1,4,5,6*]

1: Department of Neurosurgery, Mayo Clinic, Rochester, MN, USA
2: Center for Magnetic Resonance Research, Department of Radiology, University of Minnesota, MN, USA
3: Department of Physiology and Biomedical Engineering, Mayo Clinic, Rochester, MN, USA
4: Department of Radiology, Mayo Clinic, Rochester, MN, USA
5: Department of Neurology, Mayo Clinic, Rochester, MN, USA
6: Department of Physiology, College of Medicine, Hanyang University, Seoul, South Korea

*** Corresponding Author:**
Hang Joon Jo, Ph.D.
E-mail: hangjoonjo@hanyang.ac.kr
Department of Physiology, College of Medicine, Hanyang University, Seoul, Korea

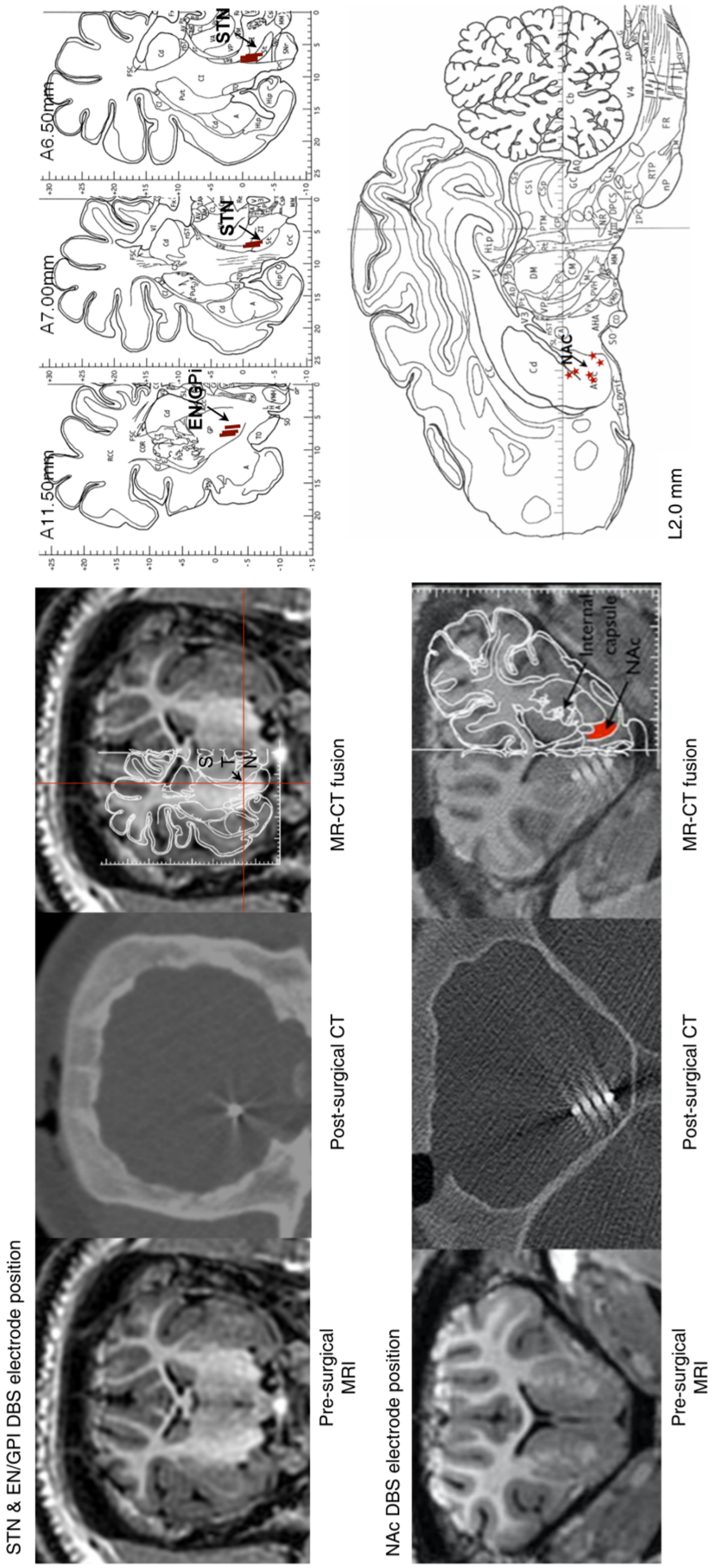

Abbreviations: CT, Computed tomography; DBS, Deep brain stimulation; EN, Entopeduncular nucleus; GP, Globus pallidus; GPi, Globus pallidus interna; HP, Hippocampus; MB, Mammillary body; MRI, Magnetic resonance imaging; STN, Subthalamic nucleus; ZI, Zona incerta

Fig. S1. DBS target confirmation in post-surgical CT

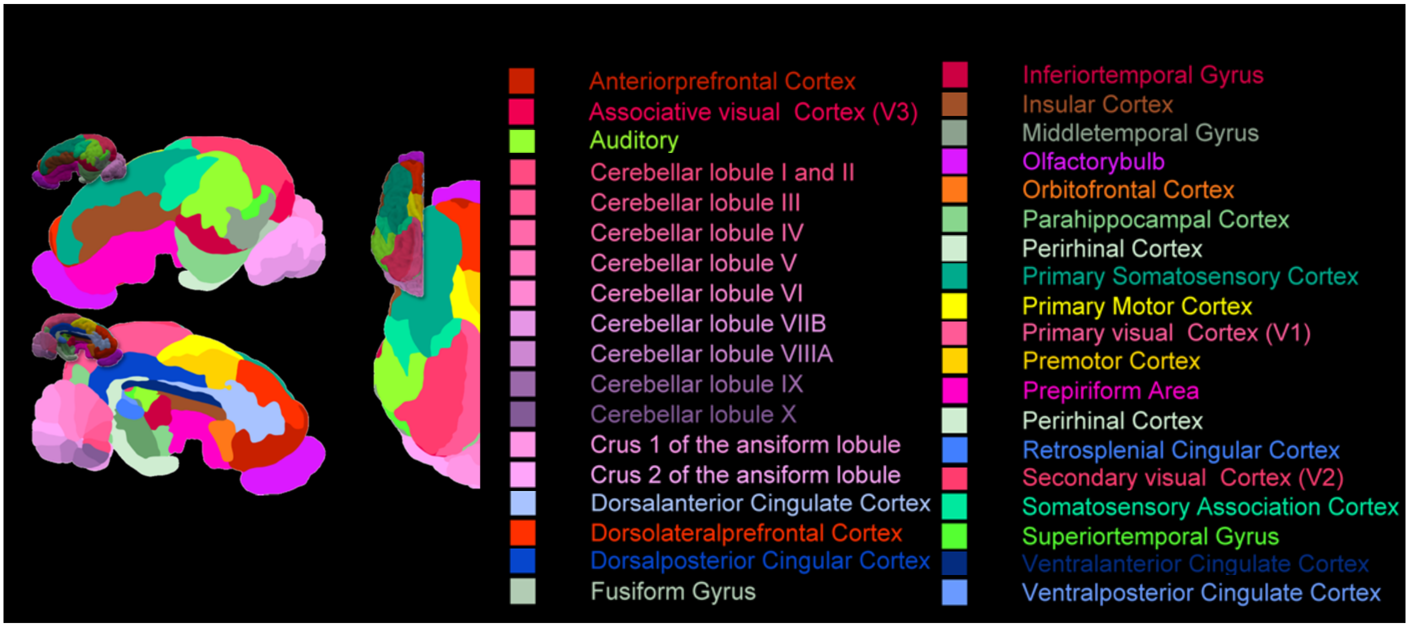

Fig. S2. Region-of-interests (ROI) key map

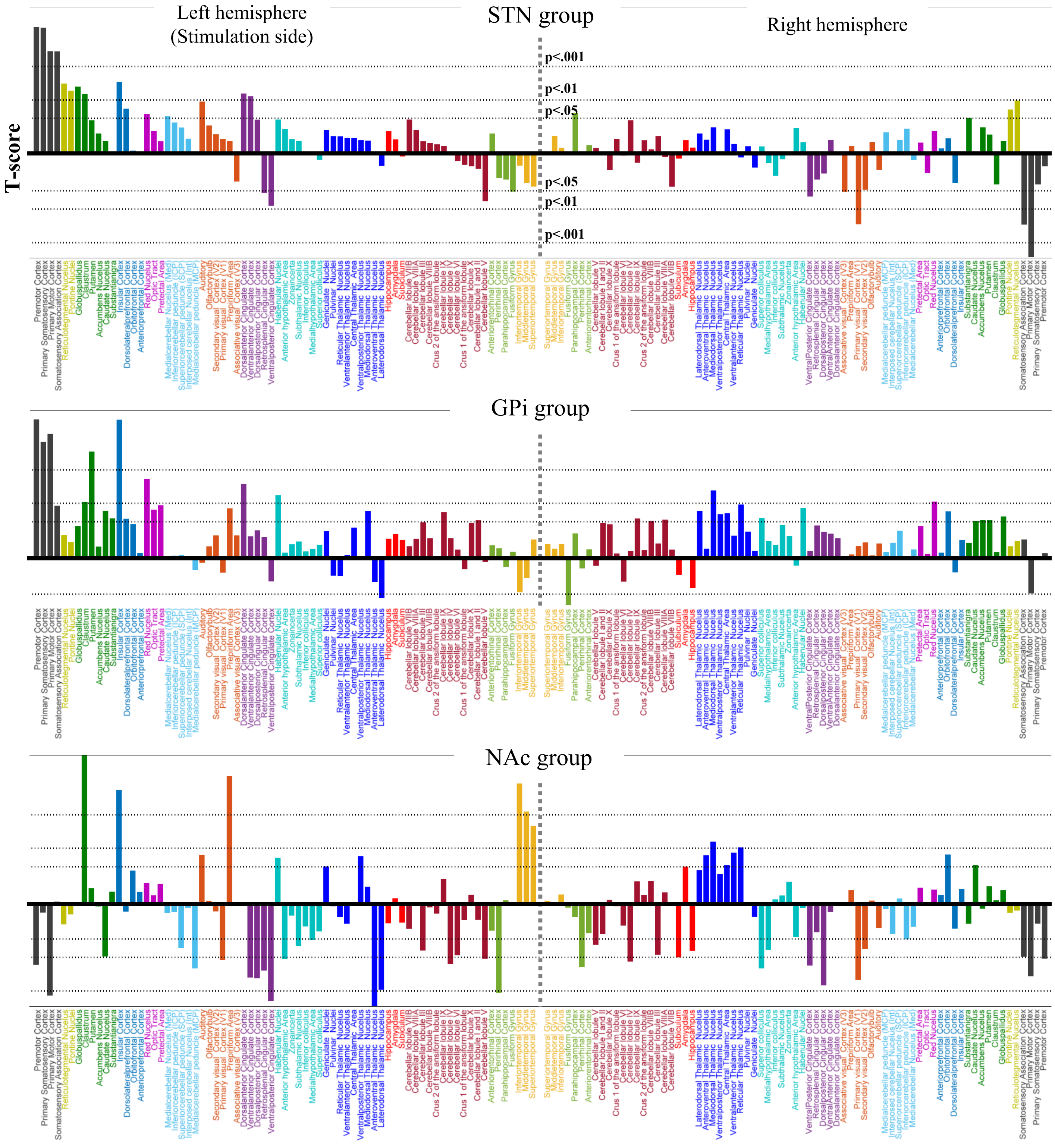

Fig. S3. The *t*-statistics of BOLD signal change in individual brain regions, STN DBS group (top), GPi DBS group (middle), and NAc DBS group (bottom). The horizontal dotted lines indicate the statistical significance level (*p*-value) for $p < .05$, $p < .01$, and $p < .001$ from one-sample two-tail *t*-test. Note that electrodes were implanted in the left hemisphere.

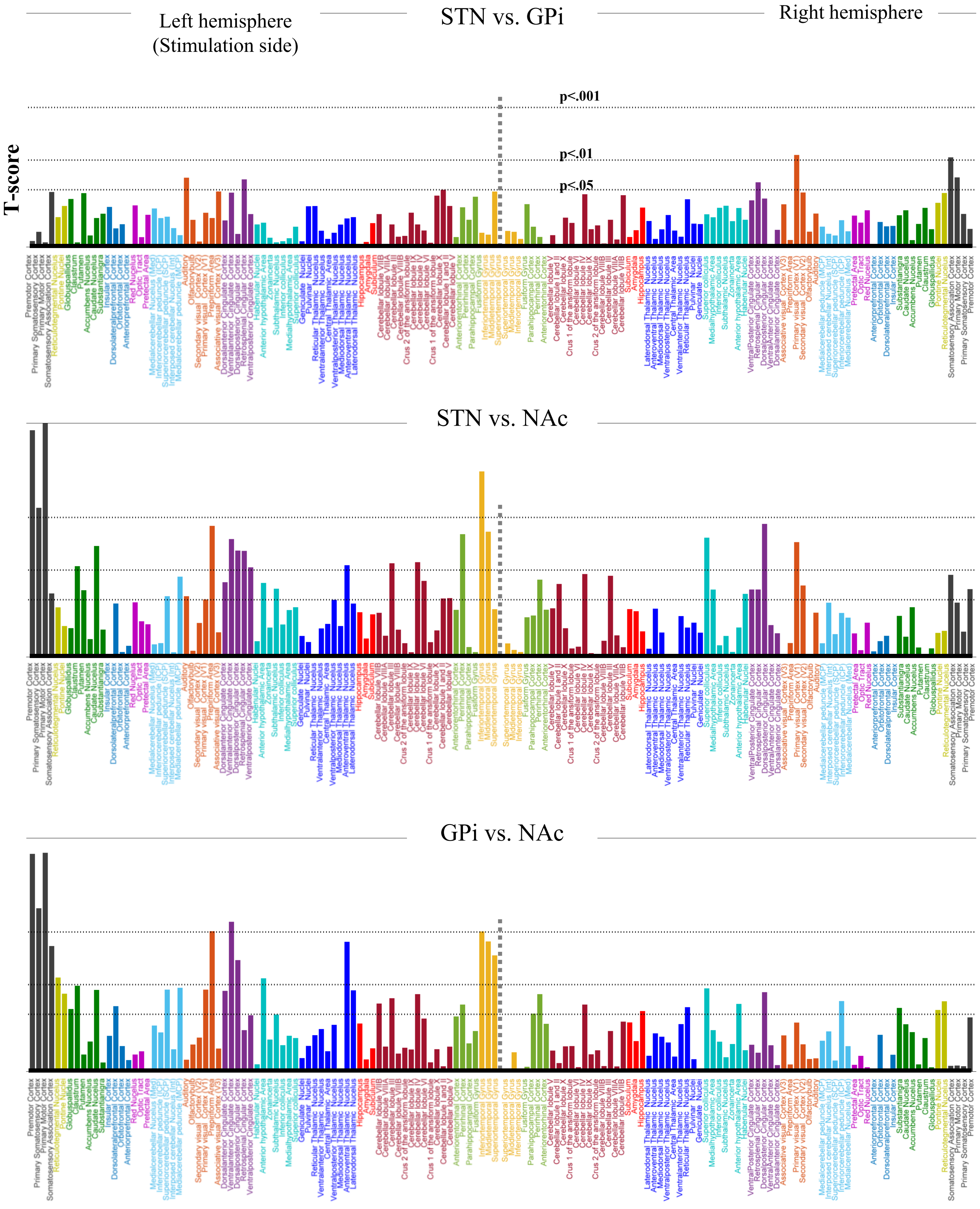

Fig. S4. The *t* scores of each brain region showing the significance of BOLD signal changes between two groups: STN versus GPi, STN versus NAc, and GPI versus NAc. The horizontal dotted lines indicate the significance level (p-value) for p<.05 , .01, and .001. Note that electrodes were implanted in the left hemisphere.

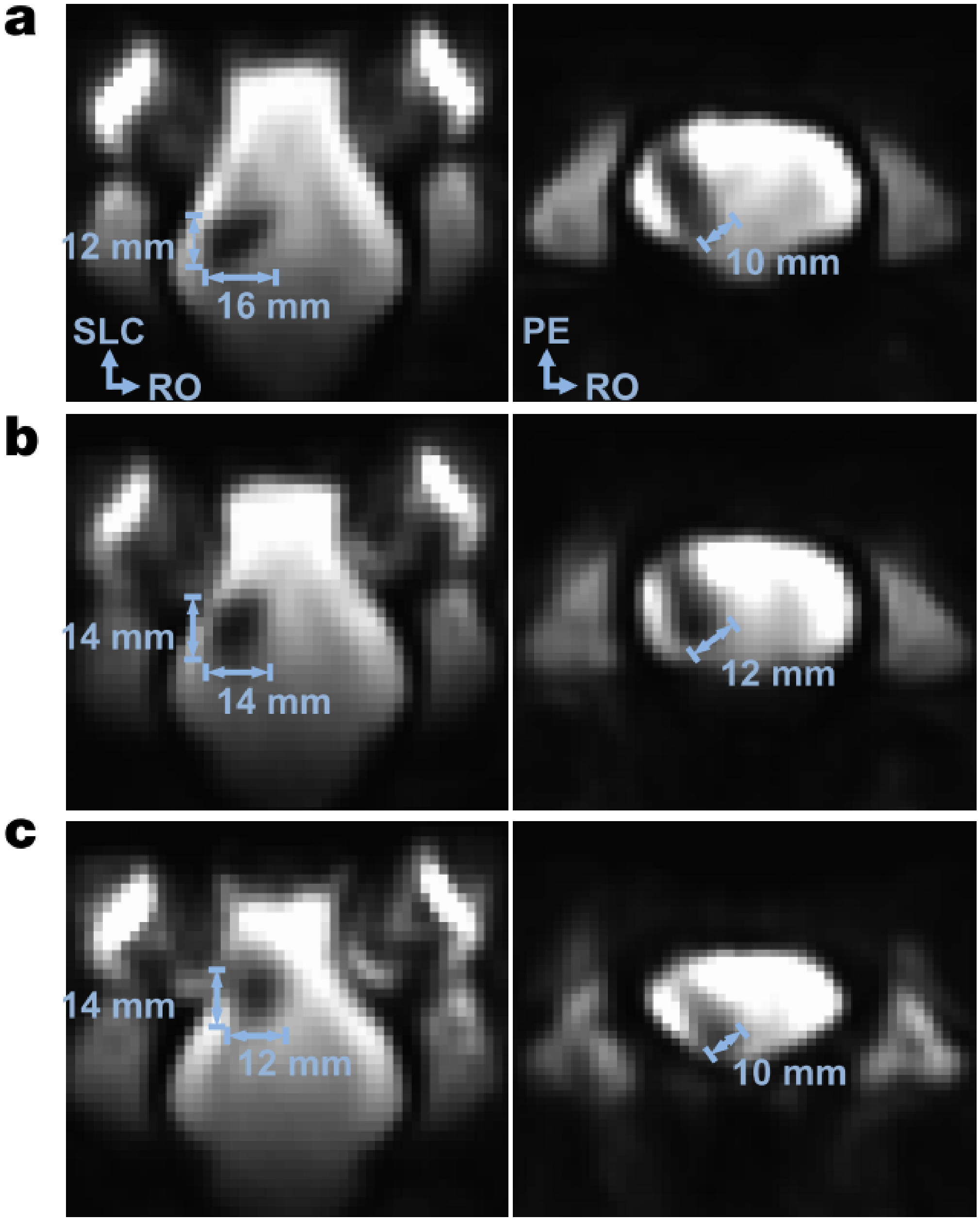

Fig. S5. Susceptibility artefact of DBS electrode lead noted in gradient-echo echo-planar imaging (7 subjects averaged per group) for (A) STN, (B) GPi, and (C) NAc DBS group. The width and length of the signal drop out area in EPI images was measured; slice (SLC), read-out (RO), and phase encoding (PE) direction indicated by arrows.
